# Sound localization with head movement: implications for 3-d audio displays

**DOI:** 10.3389/fnins.2014.00210

**Published:** 2014-08-12

**Authors:** Ken I. McAnally, Russell L. Martin

**Affiliations:** Aerospace Division, Defence Science and Technology OrganisationMelbourne, VIC, Australia

**Keywords:** audio displays, sound localization, auditory-vestibular integration

## Abstract

Previous studies have shown that the accuracy of sound localization is improved if listeners are allowed to move their heads during signal presentation. This study describes the function relating localization accuracy to the extent of head movement in azimuth. Sounds that are difficult to localize were presented in the free field from sources at a wide range of azimuths and elevations. Sounds remained active until the participants' heads had rotated through windows ranging in width of 2, 4, 8, 16, 32, or 64° of azimuth. Error in determining sound-source elevation and the rate of front/back confusion were found to decrease with increases in azimuth window width. Error in determining sound-source lateral angle was not found to vary with azimuth window width. *Implications for 3-d audio displays*: the utility of a 3-d audio display for imparting spatial information is likely to be improved if operators are able to move their heads during signal presentation. Head movement may compensate in part for a paucity of spectral cues to sound-source location resulting from limitations in either the audio signals presented or the directional filters (i.e., head-related transfer functions) used to generate a display. However, head movements of a moderate size (i.e., through around 32° of azimuth) may be required to ensure that spatial information is conveyed with high accuracy.

Three-dimensional (3-d) audio displays are designed to create an illusion of immersion in an acoustic environment by presenting via headphones the acoustic signals that would normally be present at a listener's ears (Wightman and Kistler, 1989). It has been proposed that such displays be included in a number of work environments, for example aviation (Begault, 1998), where spatial information could be imparted to operators by the direction of virtual acoustic sources. For virtual sound sources to appear stable in the world, the position and orientation of the listener's head must be tracked and head movement compensated for by updating the head-referenced, head-related transfer functions (HRTFs) that render virtual acoustic space.

There are at least three issues that may limit the utility of a 3-d audio display of directional information. The first is that listeners commonly mislocalize sounds to the incorrect front/back hemifield (Oldfield and Parker, 1984) and the rate of these errors is generally higher when listening to a 3-d audio display than when listening in the free field (e.g., Wightman and Kistler, 1989). The second is that spectral cues to source location (Shaw and Teranishi, 1968; Blauert, 1969/1970) are highly listener specific (Wenzel et al., 1993) and care must be taken to reproduce these cues accurately to ensure good localization performance. This may require the measurement of HRTFs for each individual listener. The third is that not all sounds can be well localized. For a sound to be well localized, it must have a broad bandwidth and a relatively flat spectrum that does not mask monaural spectral cues to location (King and Oldfield, 1997).

Cues to sound-source location also include interaural differences in the time of arrival (the interaural time difference, ITD) and level (the interaural level difference, ILD) of a sound. These cues are ambiguous and, to a first approximation, specify a cone-of-confusion centered on the interaural axis upon which a source lies (e.g., Mills, 1972). Monaural spectral cues resulting from the interaction of a sound wave with the external ear, head and torso can be used to specify the source elevation and front/back hemifield (see Carlile et al., 2005, for a review).

Wallach (1940) suggested that dynamic ITDs and ILDs associated with movement of the head should resolve confusion regarding the front/back hemifield of a sound source. Using speakers located in front of a listener, Wallach was able to simulate sources in the rear by manipulating the direction in which ITDs and ILDs changed as a listener's head rotated in azimuth. Macpherson (2013) has since shown that it is dynamic ITDs rather than ILDs that provide a strong cue to front/back hemifield. The role of head movement in resolving front/back confusion has also been confirmed by other studies in which the head was allowed to move during signal presentation (Thurlow et al., 1967; Perrett and Noble, 1997a; Wightman and Kistler, 1999; Iwaya et al., 2003). However, in many of these studies (Perrett and Noble, 1997a; Wightman and Kistler, 1999; Iwaya et al., 2003; Macpherson, 2013) confusions were not entirely eliminated by head movement.

Wallach (1940) also suggested that the rates of change of ITDs and ILDs with changes in head azimuth would provide a cue to sound-source elevation. ITDs and ILDs change most rapidly with changes in head azimuth when sources are on the horizon. For sources directly above or below a listener, they are unaffected by head azimuth. Wallach was able to simulate sound sources at different elevations by manipulating the rate at which the sound source was switched from one location on the horizon to another as the listener's head rotated in azimuth.

That head movement can improve localization in elevation has been confirmed by a number of subsequent studies (Thurlow and Runge, 1967; Perrett and Noble, 1997a,b; Kato et al., 2003). In one of those studies, Perrett and Noble (1997b) showed that dynamic ITD cues can compensate for the disruption of monaural spectral cues that results when tubes are inserted into the ear canals. Similarly, Kato et al. (2003) reported that head movement improves elevation localization when monaural spectral cues are disrupted by ear molds. These results suggest that dynamic interaural difference cues associated with head movement may compensate, at least in part, for the compromised spectral cues likely to be provided by 3-d audio displays generated using imperfect HRTFs.

Previous research, therefore, suggests that localization of sounds presented via 3-d audio displays may be improved by allowing listeners the opportunity to move their heads. While the previously described studies demonstrate that head movement can reduce the incidence of front/back confusion and the magnitude of elevation errors, the function relating sound localization accuracy to the extent of head movement has not been described. If large head movements are required to extract accurate directional information from a 3-d audio display, the display's utility would be limited in many situations, for example where operators are required to perform simultaneous visual tasks. The present study addresses this issue by examining the effect on localization accuracy of the availability of dynamic ITD and ILD cues associated with rotation of the head through windows ranging in width from 2 to 64° of azimuth. In order to simulate conditions where the HRTFs used to render a display are not of high fidelity and/or the sound to be localized has not been optimized for localization, monaural cues to sound-source elevation and front/back hemifield were reduced by randomizing the signal spectrum from trial to trial. The study was conducted in the free field, rather than a virtual acoustic environment, to ensure that the localization accuracy observed was not dependant on limitations in the fidelity of a particular 3-d audio display. In particular, it was desirable that the dynamic interaural difference cues made available by head movement were of high fidelity, and not limited by the quality of spatial interpolation between measured HRTFs.

## Methods

### Participants

Eight volunteers (six men and two women) participated. Their average age was 34.5 years. All participants had normal hearing sensitivity (i.e., their absolute thresholds were no more than one standard deviation above age-relevant norms (Corso, 1963; Stelmachowicz et al., 1989) for seven pure tones ranging in frequency from 0.5 to 16 kHz). They also had substantial experience in localizing sound within the experimental setting. All participants gave informed consent.

### Design

Head movement was allowed in six conditions, in each of which the offset of the sound to be localized was triggered when the participant's head had rotated through a predefined window of azimuth. The width of this window was 2, 4, 8, 16, 32, or 64°, as measured using a head-worn magnetic-tracker receiver (Polhemus, 3Space Fastrak). The head tracker had an accuracy of 0.08 cm in translation and 0.15° in rotation. Each participant completed two sessions, each of 42 trials, for each of the six conditions. The order of conditions followed a randomized-blocks design.

### Stimulus generation

The sound to be localized was broadband noise with a spectrum that varied randomly from trial to trial to reduce monaural spectral cues to source elevation and front/back hemifield. All stimuli were generated digitally at 50 kHz (Tucker-Davis Technologies system II). The spectrum of each random-spectrum noise comprised 42 bands centered on frequencies ranging from 0.013 to 19.7 kHz. The width of each band was one equivalent rectangular bandwidth (Glasberg and Moore, 1990). The level of each band was set to a random value within a 60-dB range. Rise and fall times were 40 ms. Stimuli were passed through a digital filter that compensated for variations in the response of the loudspeaker through which they were presented (Bose, FreeSpace tweeter) across the frequency range from 200 Hz to 20 kHz and were presented in the free field at about 65 dB SPL (A-weighted).

### Localization procedure

Participants sat on a swiveling chair in an anechoic chamber at the center of rotation of a motorized hoop on which the loudspeaker was mounted. The hoop allowed the loudspeaker to be placed at any azimuth and at any elevation between −50 and +80° with 0.1° accuracy. Their view of the loudspeaker was obscured by an acoustically transparent cloth sphere. Participants wore a headband upon which the magnetic-tracker receiver and a laser pointer were mounted. To begin each trial the participant placed his/her chin on a rest and oriented toward a light emitting diode at 0° of azimuth and elevation. When he/she pressed a hand-held button, the head's position and orientation were recorded. A stimulus was presented if the head was stationary and in the center of the hoop. Upon presentation of the stimulus, the participant was instructed to remove his/her chin from the rest and to turn his/her head and body in a direct manner in order to point the head-mounted laser pointer's beam at the location on the surface of the cloth sphere where he/she had perceived the sound source to be and then to press a hand-held button. Inspection of head motion trajectories confirmed that listeners complied with the instruction to orient directly to the perceived source. The azimuth and elevation of the location on the cloth sphere illuminated by the laser pointer, referenced to the center of the hoop, were calculated. The head's position and orientation were recorded at 25 Hz throughout each trial. No feedback was given with regard to localization performance.

Stimuli were presented from locations ranging from −180 to +180° of azimuth and from −50 to +80° of elevation. The location for any given trial was chosen pseudorandomly such that sound-source locations were distributed more-or-less evenly across the part-sphere in any given session. The loudspeaker was moved to a random location between successive trials so that the participant could not discern the sound-source location by listening to the motors controlling the hoop.

### Data analysis

Data analysis was restricted to trials in which the perceived azimuth was outside of the azimuth window. This was to ensure that the head had rotated through the desired range of azimuths and stimulus offset had been triggered. Analysis was also restricted to source locations with absolute azimuths greater than 64° in order to ensure that the distribution of sources was well matched across azimuth window conditions. These restrictions resulted in an average of 436 trials/condition (range from 432 to 440).

The proportion of trials in which a front/back confusion was made was calculated for each participant and condition. For a response to be considered a front/back confusion, the actual and perceived sound-source locations had to be in different front/back hemispheres and more than a criterion angle of azimuth (i.e., 7.5° divided by the cosine of the location's elevation to adjust for convergence of azimuth at the poles) from the plane separating the front and back hemispheres. Trials in which actual and/or perceived sound-source locations were close to that plane were excluded when calculating front/back confusion rate because it could not be concluded with confidence that a front/back confusion had occurred where this was the case. Localization errors comprising unsigned errors of lateral angle and elevation were calculated for all responses that were not classified as front/back confusions. Lateral angle is defined as the angle between a source and the median plane and indicates the cone of confusion upon which the source lies. Elevation is defined as the angle between a source and the interaural horizontal plane.

Results were analyzed using either one-way, repeated-measures analyses of variance (ANOVAs), incorporating Greenhouse–Geisser corrections for violations of the assumption of sphericity where appropriate (e.g., Keppel, 1991), or Friedman analyses of variance by ranks. Post-hoc comparisons were made using either paired-sample *t*-tests or Wilcoxon tests, correcting for the false discovery rate (Benjamini and Hochberg, 1995). Significant effects were further explored by regression analyses. An *a priori* alpha level of 0.05 was applied when interpreting all inferential statistics.

## Results

**Figure 1:**
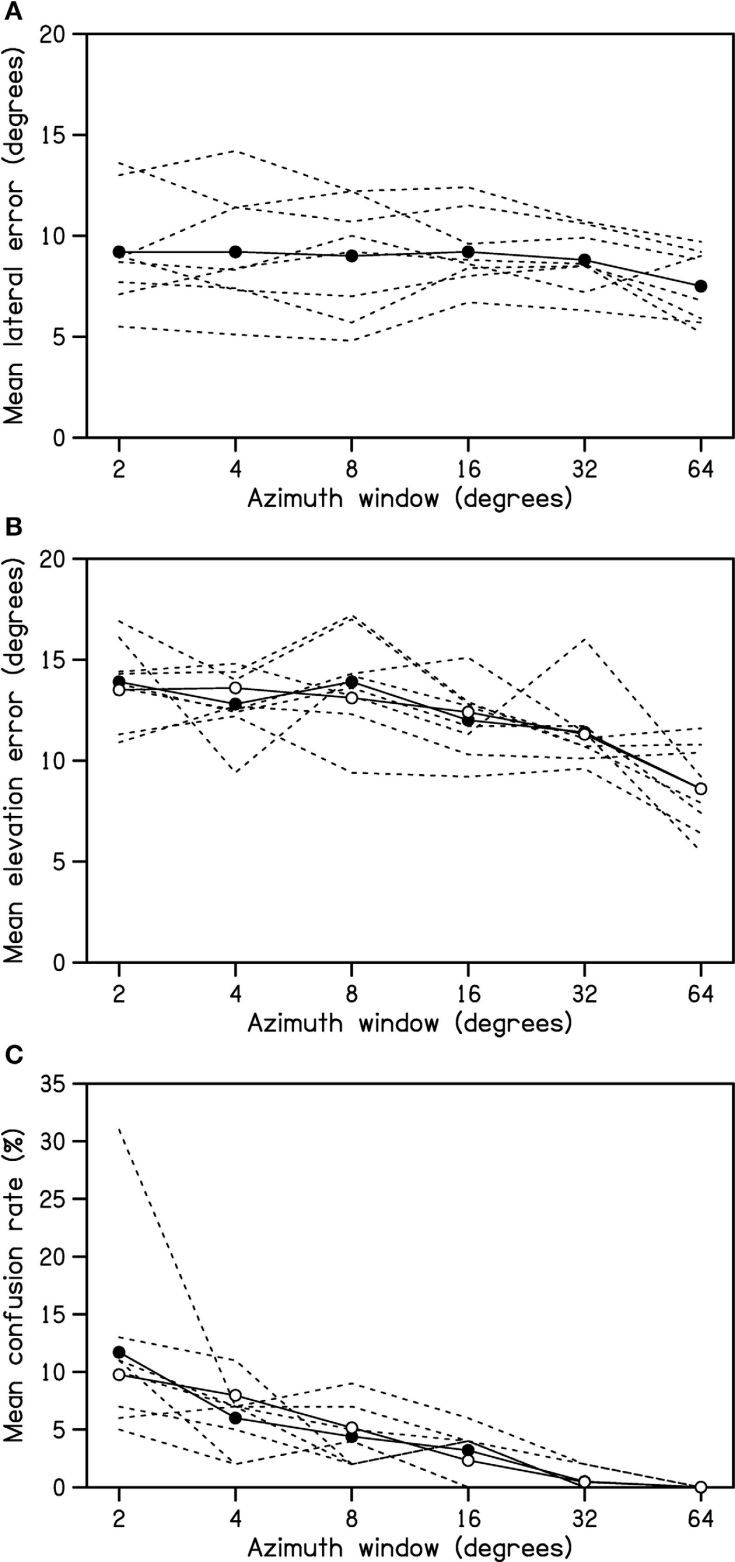
**Mean lateral error (A), elevation error (B), and front/back confusion rate (C) as functions of azimuth window width**. Dashed lines represent means for individual participants. Solid symbols with solid lines represent means across participants. Open symbols with solid lines represent means predicted by the regression, partialling out the effects of duration and the extent of head rotation in elevation.

Mean lateral errors for individual participants, and averaged across participants, are shown in Figure 1A. A One-Way repeated-measures ANOVA revealed that the effect of azimuth window was not significant, *F*_(3.1, 21.9)_ = 2.63, *p* = 0.07, partial η^2^ = 0.27.

Mean elevation errors for individual participants, and averaged across participants, are shown in Figure 1B. The overall mean elevation error was around 14 for the 2° azimuth window which confirms that spectral cues to elevation were reduced. [The mean elevation error is normally around 8° for a brief white noise stimulus (Martin et al., 2004)]. A One-Way repeated-measures ANOVA revealed a significant effect of azimuth window, *F*_(3.0, 21.1)_ = 10.8, *p* < 0.001, partial η^2^ = 0.61. *Post-hoc* comparisons, controlling for the false discovery rate, revealed that mean elevation error for the 2° azimuth window was significantly larger than those for all windows greater than 8°, and that the mean elevation error for the 64° window was significantly smaller than those for all windows less than 32°, *t*_(7)_ ≥ 2.92, *p* ≤ 0.02.

Mean rates of front/back confusion, shown in Figure 1C, decreased to zero with increasing azimuth window width. A Friedman analysis of variance by ranks revealed that the effect of azimuth window was significant, χ^2^_(5)_ = 34.4, *p* < 0.001. *Post-hoc* Wilcoxon tests, controlling for the false discovery rate, revealed that all comparisons were significant with the exception of 4 vs. 8°, 8 vs. 16°, and 32 vs. 64°, *Z* ≥ 2.33, *p* ≤ 0.02.

In addition to the extent of head rotation in azimuth during stimulus presentation, the width of the azimuth window could be expected to be correlated with both stimulus duration and the extent of head rotation in elevation^1^. That this was the case is confirmed by the data presented in Table 1, which show significant correlations between stimulus duration, the extent of head rotation in azimuth, and the extent of head rotation in elevation during signal presentation. It is therefore unclear which of these three variables was responsible for the above-described effects of azimuth window on elevation error and front/back confusion rate.

**Table 1:** **Pearson correlations between stimulus duration and the extents of head rotation in azimuth and elevation during stimulus presentation**.

	**Azimuth rotation**	**Elevation rotation**
Duration	0.24	0.37
Azimuth rotation		0.57

Note: All p-values < 0.001.

In order to determine which of these variables influenced elevation error, a multiple regression analysis was conducted. Stimulus duration, the extent of head rotation in azimuth, and the extent of head rotation in elevation were the predictor variables of interest. To facilitate the interpretation of relationships between elevation error and these variables, the absolute lateral angle and elevation of the sound source and the individual participant were added to the predictor variable list.

The complete regression model was found to explain 16.9% of the observed variance in elevation error. As shown in Table 2, all three predictors of interest explained a significant, unique component of this variance. Elevation error was found to decrease significantly with increasing head rotation in either azimuth or elevation. Of some surprise, elevation error was found to *increase* significantly with increasing stimulus duration. The mean elevation errors predicted by the regression, partialling out the effects of duration and the extent of head rotation in elevation, are plotted in Figure 1B (open symbols) and follow a similar form to the raw means.

**Table 2:** **Results of multiple regression predicting elevation errors for trials where a front/back confusion was not made**.

**Predictor**	***β***	***t***	***p***
Participant 1	0.042	1.75	0.08
Participant 2	−0.001	−0.03	0.97
Participant 3	0.027	1.14	0.25
Participant 4	−0.031	−1.27	0.20
Participant 5	0.014	0.54	0.59
Participant 6	0.025	1.02	0.30
Participant 7	0.021	0.89	0.37
Source |lateral angle|	−0.127	−6.05	<0.001
Source |elevation|	0.298	13.89	<0.001
Duration	0.104	4.37	<0.001
Azimuth rotation	−0.111	−4.82	<0.001
Elevation rotation	−0.096	−3.86	<0.001

A multiple logistic regression analysis predicting front/back confusion rate from the same list of variables was conducted to determine which of the three predictors of interest influenced this error measure. The complete logistic regression model was found to explain 26.3% of the observed variance in front/back confusion rate. As shown in Table 3, both stimulus duration and the extent of head rotation in azimuth explained a significant, unique component of this variance. Front/back confusion rate was found to increase significantly with increasing stimulus duration (odds ratio > 1) and decrease significantly with increasing head rotation in azimuth (odds ratio < 1). The extent of head rotation in elevation was found to have no significant unique influence on front/back confusion rate. The mean front/back confusion rates predicted by the regression, partialling out the effects of duration and the extent of head rotation in elevation, are plotted in Figure 1C (open symbols) and follow a similar form to the raw means.

**Table 3:** **Results of multiple logistic regression predicting front/back confusion rates**.

**Predictor**	**Odds ratio**	**Wald**	***p***
Participant 1	0.57	1.05	0.30
Participant 2	0.15	15.64	<0.001
Participant 3	0.59	0.92	0.34
Participant 4	0.58	1.01	0.31
Participant 5	0.23	8.35	0.004
Participant 6	0.29	6.02	0.014
Participant 7	0.39	3.35	0.07
Source |lateral angle|	1.02	7.66	0.006
Source |elevation|	1.05	50.01	<0.001
Duration	1.22	3.76	0.05
Azimuth rotation	0.89	38.34	<0.001
Elevation rotation	1.02	0.85	0.36

## Discussion

In many situations where a 3-d audio display could be applied it may not be possible or desirable for a listener to freely move his/her head in order to enhance sound localization. For example, the range of desirable head movements may be limited by concurrent visual tasks in the work environment. In order to predict the localization performance that can be expected in different situations, it is necessary to understand the manner in which the accuracy of sound localization varies as a function of the extent of head movement. The present study describes the function relating localization errors to the extent of head movement in greater detail than does any previous study. The extent of head movement was constrained by terminating the auditory stimulus when the participant's head had rotated through a predefined window of azimuth, and the nature of head movement was constrained by instructing the participant to orient directly toward the perceived location of the sound source. Head rotation through an azimuth window as narrow as 4° was found to significantly reduce the rate at which front/back confusions were made. In contrast, head rotation through an azimuth window of 16° was found to be required to significantly reduce elevation error. Head movement was not found to significantly affect lateral localization error.

Most previous studies of the effect of head movement on sound localization allowed free head movement, and controlled neither the range nor the manner of that movement (Thurlow and Runge, 1967; Perrett and Noble, 1997a; Wightman and Kistler, 1999; Iwaya et al., 2003; Kato et al., 2003). Although some previous studies included a condition in which the range of movement was constrained by verbal instruction, for example to between −30 and +30° of azimuth or to between two light emitting diodes (Perrett and Noble, 1997a,b), the small number of movement conditions in those studies does not allow a description of the function relating localization performance to the extent of head movement during stimulus presentation. In a recent study by Macpherson and Kerr (2008), sound onset and offset were gated with reference to head azimuth across a range of window widths. However, that study only examined localization in azimuth for sources on the horizon. The present study extended Macpherson and Kerr's study by examining localization in azimuth and elevation as well as front/back confusion for sources distributed across most of the sphere.

In this study, larger movements of the head were associated with longer stimulus durations. For example, the mean stimulus duration for the 2° azimuth window was 1.2 s, whereas that for the 64° azimuth window was 1.8 s. However, as we observed that stimulus duration was *positively* related to both elevation error magnitude and the rate of front/back confusion, it seems unlikely that the reductions in mean elevation error and front/back confusion rate that accompanied increases in azimuth window width were driven by the associated increases in stimulus duration. Rather, they appear to be attributable to the associated increases in the extents of head rotation during stimulus presentation. The fact that we found no evidence to indicate that localization accuracy improves as stimulus duration increases beyond a second or so is consistent with previous studies that have shown that functions relating localization or lateralization performance and stimulus duration are asymptotic at durations considerably less than one second (Tobias and Zerlin, 1957; Hofman and van Opstal, 1998). For example, Hofman and van Opstal inferred that the auditory localization system can form a stable estimate of sound-source azimuth and elevation on the basis of a sample of about 80 ms. The positive relationship we observed between stimulus duration and localization errors may be attributable to a tendency for participants to orient more slowly toward stimuli which were difficult to localize.

It has commonly been reported that although head movement reduces the incidence of front/back confusion, it does not necessarily eliminate such confusion (Perrett and Noble, 1997a; Wightman and Kistler, 1999; Iwaya et al., 2003; Macpherson and Kerr, 2008). For example, Macpherson and Kerr (2008) examined the effect of head rotation through 0, 2.6, 5, and 20° azimuth windows at a rate of 50°/s on localization in azimuth for sources of wide-band noise, low-frequency noise, and high-frequency noise. Low-frequency noise is the most comparable of their stimuli to the random-spectrum noise used in the present study because it would have provided robust interaural time difference (ITD) cues, including dynamic ITD cues, but poor monaural spectral cues to location. In the case of that stimulus, Macpherson and Kerr (2008) observed a marked reduction in the rate of front/back confusion for azimuth windows as narrow as 2.6°, but windows of around 20° were required to eliminate these confusions. In the present study, head movement through a 4° azimuth window significantly reduced the front/back confusion rate, but movement through a 32° azimuth window was required to almost eliminate these confusions.

Wallach (1940) showed that the rate of change of interaural difference cues with head rotation in azimuth provides a cue to (the absolute value of) a sound source's elevation. This is because the elevation of a source determines the rate at which its lateral angle changes as the head is rotated in azimuth. The negative relationship we observed between elevation error magnitude and the extent of head rotation in azimuth during stimulus presentation is thus consistent with Wallach's (1940) proposal that dynamic interaural difference cues are integrated with knowledge about head rotation in azimuth to help determine sound-source location. It can be seen from Figure 1 that all listeners were similarly able to integrate vestibular and/or proprioceptive information about head rotation with dynamic auditory cues to improve sound localization.

The negative relationship we observed between elevation error magnitude and the extent of head rotation in elevation during stimulus presentation, in contrast, is suggestive of the presence of *dynamic spectral cues* to sound-source elevation. That is, it suggests that the way in which a sound's spectra at the ears changes as the head is rotated in elevation provides information concerning the elevation of its source. Because the source spectrum was constant within a trial, any such dynamic spectral cues would not be expected to be disrupted by the trial-to-trial spectral roving that was applied to the stimuli in order to reduce static spectral cues to source location.

In order to determine whether the information concerning head movement that participants integrate with dynamic interaural difference cues to refine localization judgements is derived from vestibular or proprioceptive sources, Kim et al. (2013) compared azimuthal sound localization under conditions of active head movement, passive head movement, and body movement with the head fixed. They concluded that vestibular information associated with head movement is both necessary and sufficient to improve sound localization. In contrast, they found that proprioceptive information does not improve localization.

We expect that the observed beneficial effects of head movement on sound localization will generalize to (i) other situations where audio signals (e.g., warnings) have spectra that are not optimal for localization and (ii) situations where a 3-d audio display is generated using imperfect HRTFs, such as those that have not been tailored for the particular listener. While small head movements were found to reduce the rate of front/back confusion, moderate movements (i.e., around 16–32°) were found to be required to significantly reduce elevation errors and to almost eliminate confusions. In situations where head movements of this magnitude are impractical, it will be necessary to optimize both the HRTFs used to generate a 3-d audio display and the signals presented through it in order to ensure that the display is of high spatial fidelity.

### Conflict of interest statement

The authors declare that the research was conducted in the absence of any commercial or financial relationships that could be construed as a potential conflict of interest.

## Footnotes

^1^It is important to note that the actual extent of head rotation in azimuth during stimulus presentation was not completely determined by the width of the azimuth window for two reasons. First, the azimuth windows were symmetric about the midline. Participants occasionally rotated their heads a little away from the source when exiting the chin rest. Second, stimulus offset was triggered when the head had rotated through the azimuth window but occurred with a slight delay because of the low sample rate of the head tracker. For these reasons, our regression analyses utilized the actual extent of head rotation in azimuth during signal presentation rather than azimuth window width. The extent of head rotation in azimuth was defined as the range of azimuth through which the head rotated during signal presentation. Similarly, the extent of head rotation in elevation was defined as the range of elevation through which the head rotated during signal presentation.
